# A Viral Ecogenomics Framework To Uncover the Secrets of Nature’s “Microbe Whisperers”

**DOI:** 10.1128/mSystems.00111-19

**Published:** 2019-05-14

**Authors:** Simon Roux

**Affiliations:** a DOE Joint Genome Institute, Walnut Creek, California, USA

**Keywords:** ecogenomics, metagenomics, phage, virus

## Abstract

Microbes drive critical ecosystem functions and affect global nutrient cycling along with human health and disease. They do so under strong constraints exerted by viruses, which shape microbial communities’ structure and shift host cell metabolism during infection. While the majority of viruses and their associated impacts remain poorly characterized, a number of mechanisms by which viruses alter microbial cells and ecosystems have already been revealed. Here I outline how a comprehensive host-resolved mapping of viral sequence space will enable a thorough characterization of virus-encoded mechanisms for microbial manipulation. With soon-to-be millions of virus genomes obtained from metagenomes, one of the major challenges resides in the development of methods for high-throughput and high-resolution virus-host pairing, before multi-omics approaches can be leveraged to fully decipher virus-host dynamics in nature. Beyond novel fundamental biological knowledge, these studies will likely provide new molecular tools enabling a precise engineering of microbial cells and communities.

## Abstract (precis)

Microbes drive critical ecosystem functions and affect global nutrient cycling along with human health and disease. They do so under strong constraints exerted by viruses, which shape microbial communities’ structure and shift host cell metabolism during infection.

## PERSPECTIVE

Microbes are now recognized to play key roles in all of Earth’s ecosystems, driving nutrient and energy transfers, and directly influencing human health and disease (1). These microbial processes are strongly constrained by viruses, which are globally abundant and infect all cellular life forms. Because of technical challenges limiting our ability to cultivate viruses infecting these microbes and, until recently, to even survey their diversity in nature, the vast majority of viruses and their associated impacts remain poorly understood. Nevertheless, a number of mechanisms by which viruses profoundly influence microbial ecosystems have already been revealed or predicted (2, 3). The most intuitive impact of viral infection is linked to virus-induced mortality which can trigger large-scale reshuffling of microbial communities, although the associated ecological and evolutionary drivers are not fully characterized yet. However, viruses can also modify host cell metabolism and alter host cell fitness during infection.

Modification of host cell properties during viral infection has been primarily observed and studied for temperate bacteriophages integrated into the host’s genome (prophages) (3, 4). Prophages were found to alter their host cell features through gene disruption (when integrated), protection from infection by similar and/or unrelated phages (superinfection exclusion), and introduction of new genes either through horizontal transfer or directly expressed from the prophage (lysogenic conversion). The latter notably includes virus-borne toxin genes leading to increased virulence for major bacterial pathogens such as Vibrio cholerae, Clostridium botulinum, or Escherichia coli (5). Meanwhile, the protection provided by phages against additional infections has consequences not just for individual host cells but also for the whole community dynamics by altering virus/host ratios and global infection efficiency (6). Beyond temperate phages, fitness-altering genes have been discovered in lytic viruses, i.e., viruses whose infection cycle does not include a latent phase but who instead directly replicate their genome and produce new virus particles upon entry in the host cell. Photosystem genes carried in lytic cyanophages were the first example of host cell reprogramming by lytic viruses to be comprehensively characterized (7), but other genes potentially regulating nitrogen, sulfur, phosphorus, and central carbon metabolisms have since been identified. Collectively, these studies suggest that viruses, both temperate and lytic, manipulate a broad range of host cell features.

Because exploring environmental viral communities has remained a challenging task until very recently, our collective understanding of the molecular mechanisms by which viruses can influence microbial cells is still largely partial, and known examples are mostly derived from a few well-studied cultivated model systems. Interrogating the vast diversity of uncultivated viruses holds tremendous potential for the discovery of entirely novel avenues for microbial manipulation, including strategies tailored to specific hosts and environmental conditions. Fortunately, this type of approach might become viable in a not-too-distant future considering the current pace of progress and improvements in the field of viral ecogenomics. Here I outline the major steps and remaining challenges toward a comprehensive characterization of viral diversity and virus-host interactions in nature and the potential use of this knowledge for manipulation of microbial communities.

## A GLOBAL MAP OF EARTH'S VIRUSES

In order to fully understand and leverage the impact of viruses on microbial cells, a large-scale mapping of viral sequence space covering most (if not all) of viral diversity must first be achieved. This is the topic that saw the most remarkable improvement in the last few years, especially compared to the exploration of microbial diversity, mainly due to a shift in the methods used. The vast diversity of uncultivated microbes was initially revealed through 16S and 18S amplicon sequencing (8); however, this approach cannot be applied to viruses as they lack a universally conserved gene. Hence, while amplicon-based surveys of microbial communities were comprehensive and revealed large portions of novel diversity, their viral counterparts focused on small groups of known viruses. Uncultivated organisms are, however, increasingly studied through *de novo* genome assembly from shotgun metagenomes (9). In contrast to amplicon sequencing, viruses are often more easily amenable to genome assembly from metagenomes than microbes, primarily because viruses tend to have smaller genomes, a lower repeat content, and higher levels of differentiation between genotypes (10).

**FIG 1:**
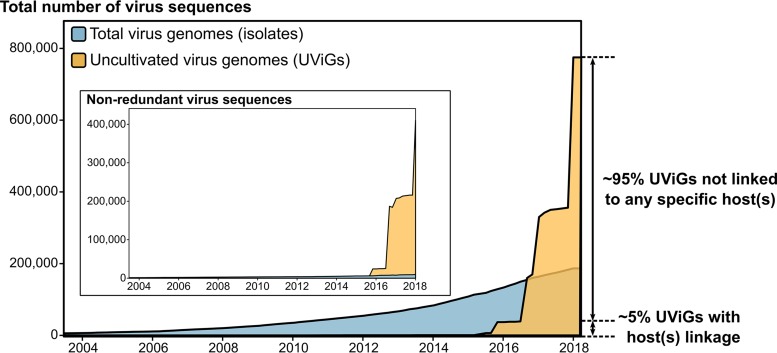
Size of virus genome databases over time. The total number of genomes from isolates was based on queries to the NCBI nucleotide database portal, while the number of uncultivated virus genomes (UViGs) was estimated by compiling data from the literature and from the IMG/VR database, as in reference 12. The inset displays the same data after dereplication of the genomes, i.e., one per species for NCBI nucleotide database, and clustering based on pairwise nucleotide similarity for UViGs using cutoffs of 95% average nucleotide identity and 85% alignment fraction (12). Host linkage information is from the IMG/VR database and was derived from matches between UViGs and isolate viruses, prophages, and CRISPR spacers.

Pragmatically, this means that the advent of genome assembly from metagenomes has been particularly impactful for the exploration of viral diversity (Fig. 1). Currently, the largest database of uncultivated virus genomes (IMG/VR; https://img.jgi.doe.gov/cgi-bin/vr/main.cgi) is composed of >700,000 uncultivated genome fragments, the overwhelming majority collected in the last 2 years alone (11). Among these, >25,000 were estimated to represent near-complete or complete genomes (≥90% complete, “high-quality viral genomes” sensu [12]). This means that metagenome assemblies have already yielded more than twice the total number of (near-)complete genomes obtained from isolates. In addition, complementary methods already exist for viruses recalcitrant to assembly from metagenomes, including viral single-amplified genomes (13) and single-template long-read sequencing (14). The pace at which data accumulate and new complementary methods are developed suggests that an exhaustive mapping of virus genome diversity is on the horizon.

## ESTABLISHING HIGH-RESOLUTION VIRUS-HOST LINKAGES

One of the main pitfalls of genome assembly from metagenomes is that, in contrast to viral isolates, uncultivated viruses are not immediately associated with a host. This host linkage information is critical to identify and interpret virus impacts on microbial ecosystems. Currently, most of this virus-host linkage is based on computational predictions using methods still being actively developed and improved, including identification of horizontal gene transfers between virus and hosts, sequence similarity between host CRISPR spacers and viral genomes, and detection of nucleotide composition (kmer) similarity between host and virus genomes (15). While these approaches are promising, they remain limited in scope and resolution. The vast majority of uncultivated virus sequences remain without any host (Fig. 1), and *in silico* predictions typically provide information on host taxonomy at the family or genus ranks, more rarely down to species, while virus-host interactions tend to be strain specific.

One of the major challenges faced by the field of viral ecogenomics is thus to develop experimental approaches providing high-throughput, high-resolution host linkage for uncultivated viruses. These approaches will most likely rely on high-throughput sequencing of single cells and/or targeted host (sub)populations, sorted and selected via a combination of, e.g., microfluidic separation, microdroplet encapsulation, and/or flow cytometry cell sorting. The codetection of virus and host at the individual cell level should then provide unique insights into strain-resolved host range and infection rates for individual virus genotypes in nature, without cultivation of either the microbial host or the virus. Given that pilot experiments are already ongoing, it seems reasonable to hope for some of these methods to be established and broadly available at some point in the next decade. The data obtained by applying these methods across a large range of biomes and ecological conditions will then enable the construction of a global host-resolved mapping of the virus sequence space.

## VIRUS-INSPIRED SOLUTIONS FOR MICROBE MANIPULATION

Finally, a global host-contextualized mapping of the virosphere would serve as a foundation for multi-omics studies aiming at deciphering virus-host interactions and dynamics in nature. Sample sets such as time series, patient cohorts, and incubations under controlled conditions, either in the laboratory or *in situ*, will be especially relevant in this context. Beyond a treasure trove of fundamental biological discoveries about ecological and evolutionary drivers of virus-host dynamics, these studies would also benefit every approach and biotechnological application leveraging microbes and microbial processes. Specifically, a comprehensive understanding of the broad range of virus-host interactions ongoing in nature would provide a fantastic resource that could be mined for new ways to precisely and predictably engineer microbial cells, including for targeted modification of specific populations within a complex microbial community.

Already, results from isolated viruses can provide a glimpse at the type of mechanisms and solutions for microbial manipulations that could be inspired by or derived from viruses. The first and most obvious application of viruses would be to kill specific microbial strains such as contaminants, either through active viruses, i.e., “phage therapy,” or by using only specific components of virus particles, e.g., tailocins. Viruses can also contain many key genes able to redirect entire metabolic pathways or to fundamentally alter host behavior and fitness, including variants of key cellular enzymes displaying unique properties as well as highly efficient transcriptional and translational regulators. Hence, a global and host-contextualized virus genome database could be used to identify a candidate gene(s) for modulating specific metabolisms or expressing artificial constructs in any target microbe. Finally, some viruses are particularly amenable to genome engineering and could thus be repurposed as custom-targeted delivery mechanisms. Members of the *Inoviridae* family, i.e., filamentous phages, are particularly interesting in this framework because they have a small genome which can be expressed in a nonhost cell and relatively simple virions whose properties could likely be modified by mutating only one or a couple of genes.

## CONCLUSION

Right now is an exciting time to be part of (or join) the young and highly dynamic field of virus ecogenomics. A lot of the infrastructure and conceptual framework is being built (12), and data pile up at an incredible rate which constantly enables new analyses at unprecedented scales. While fantastic discoveries have already been made, what is maybe even more impressive is the incredible potential of virus ecogenomics approaches to achieve a more detailed characterization of virus impacts on microbial ecosystems, of their role(s) in the evolution of life on earth, and of their potential for improving microbially based biotechnological applications. While the path and prospects outlined here will require important methodological and conceptual breakthroughs, it is nonetheless difficult not to feel a sense of awe (or even dizziness) when contemplating the extent of novel viruses that remain to be unveiled and all their intricate microbial manipulation mechanisms still to be discovered and leveraged.
